# Acceleration sensors in abdominal wall position as a non-invasive approach to detect early breathing alterations induced by intolerance of increased airway resistance

**DOI:** 10.1186/s13019-017-0658-5

**Published:** 2017-11-10

**Authors:** Thomas Breuer, Christian S. Bruells, Rolf Rossaint, Henning Steffen, Catherine Disselhorst-Klug, Michael Czaplik, Norbert Zoremba

**Affiliations:** 1 Department of Anaesthesiology, Medical Faculty, RWTH Aachen University, Pauwelsstr. 30, D-52074 Aachen, Germany; 2 Department of Intensive and Intermediate Care, Medical Faculty, RWTH Aachen University, Aachen, Germany; 3 Department of Thoracic and Cardiovascular Surgery, Medical Faculty, RWTH Aachen University, Aachen, Germany; 4 Institute for Applied Medical Engineering and Department of Rehabilitation and Prevention Engineering, RWTH Aachen University, Aachen, Germany; 5 Department of Anaesthesiology, Sankt Elisabeth Hospital, Gütersloh, Germany

**Keywords:** Acceleration sensors, Breathing patterns, Respiratory failure

## Abstract

**Background:**

Early detection of respiratory overload is crucial to mechanically ventilated patients, especially during phases of spontaneous breathing. Although a diversity of methods and indices has been established, there is no highly specific approach to predict respiratory failure. This study aimed to evaluate acceleration sensors in abdominal and thoracic wall positions to detect alterations in breathing excursions in a setting of gradual increasing airway resistance.

**Methods:**

Twenty-nine healthy volunteers were committed to a standardized protocol of a two-minutes step-down spontaneous breathing on a 5 mm, 4 mm and then 3 mm orally placed endotracheal tube. Accelerator sensors in thoracic and abdominal wall position monitored breathing excursions. 15 participants passed the breathing protocol (“completed” group), 14 individuals cancelled the protocol due to subjective intolerance to the increasing airway resistance (“abandoned” group).

**Results:**

Gradual increased respiratory workload led to a significant decrease of acceleration in abdominal wall position in the “abandoned” group compared to the “completed” group (*p* < 0.001), while these gradual accelerating changes were not observed in thoracic wall position (*p* = 0.484). Thoracic acceleration sensors did not detect any time- and group-specific changes (*p* = 0.746).

**Conclusions:**

The abdominal wall position of the acceleration sensors may be a non-invasive, economical and practical approach to detect early breathing alterations prior to respiratory failure.

**Trial registration:**

EK 309–15; by the Ethics Committee of the Faculty of Medicine, RWTH Aachen, Aachen, Germany. Retrospectively registered 28th of December 2015.

## Background

Respiratory failure manifesting as inadequate gas exchange is raised by a variety of pathophysiological alterations like airway obstruction, chest wall pathologies, muscular or innervation insufficiency, disturbance of alveolar-capillary units or cerebral pathologies. Especially after thoracic and abdominal surgery the respiratory function is hampered due to interventional changes [1, 2]. The resulting pathological breathing patterns are influenced by the workload of the respiratory system, leading to respiratory muscle dysfunction. This well-described phenomenon is determined by the duty cycle of the inspiratory muscle and the ratio of inspiratory pressure to its maximal capacity (P/P_max_) [3, 4]. Fatigue is evident by a reduced muscular force after exercise compared to prior baseline conditions. Due to the fact that the resulting force of respiratory muscles is inaccessible to direct measurements, several indirect methods have been established to predict respiratory muscle work like trans-diaphragmatic pressures, diaphragmatic sonography or the rapid shallow breathing index (RSBI) [5–7].

Breathing depends on continuous synchronized work of respiratory muscles. Especially the function of the diaphragm, as it is the main respiratory muscle, is vulnerable to attenuation [8]. This diaphragmatic dysfunction originated by increased respiratory load leads to muscular injury and disturbance of cellular homeostasis [9–11]. Due to the resulting functional diaphragmatic weakness auxiliary respiratory muscles need to be activated and thereby alter resulting breathing excursions [12].

The early detection of high respiratory workload is important for patients, especially mechanically ventilated individuals, at intensive care units during phases of spontaneous breathing. Monitoring technique should enable physician’s immediate intervention like a load-relief to prevent respiratory failure caused by diaphragmatic overload. Although a diversity of measurements and indices has been established, there is no highly specific method that is feasible in the common clinical setting to predict respiratory muscle weakness and continuously monitor patients breathing patterns [13–16].

This study aimed to evaluate motion sensors in abdominal and thoracic wall positions to detect alterations in breathing excursions. The predefined setting of gradual increasing airway resistance was used to mimic ascending respiratory workload.

Therefore, we hypothesized significant accelerations to be detected by the used motion sensors to demonstrate the feasibility of a new non-invasive technique to record breathing excursions.

## Methods

This trial was approved by the local ethical review board (EK 309–15; Ethics Committee, Faculty of Medicine, RWTH Aachen, Aachen, Germany).

### Subjects

Twenty-nine healthy human subjects men and women, with a medium age of 27,6 yr. (21–40 years) volunteered for this study. Informed consent was obtained from all subjects, mainly medical staff of the Department of Anaesthesiology, University Hospital of the RWTH Aachen, Germany.

### Clinical monitoring

The study was performed in the ICU-setting. The monitoring system (Phillips Intellivue, Eindhoven, The Netherlands) was used to register the following predefined physiological parameters. The end-tidal CO_2_ concentrations, and peak and airway occlusion pressure were monitored continuously. Breathing rate, tidal volume and airway pressures were registered using a clinical respirator (Evita 4, Draeger, Lübeck, Germany).

### Measurement of breathing excursion

**Fig. 1:**
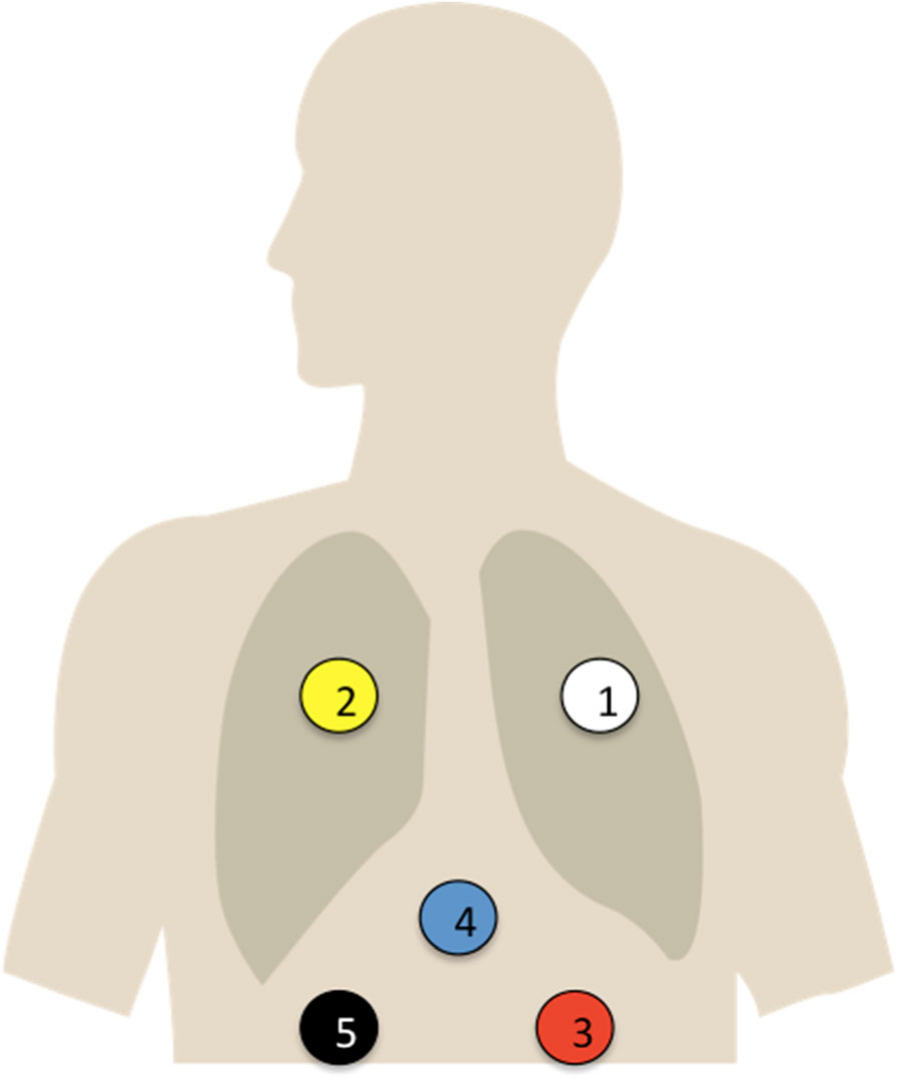
Electrode positions: Electrode 1 and 2 at the third intercostal space in the medio-clavicular line, Electrode 4 supra-umbilical in the median line and Electrode 3 and 5 bilaterally infra-umbilical next to the rectus abdominis muscle. All sensors recorded movements in three dimensions (x, y, z axis)

The three-axis-accelerometer sensors (Freescale Semiconductor Inc., Austin, Texas, USA; Model: MMA7261QT) used in this study were placed on chest and abdominal wall (See Fig. 1). The sensors were located on the third intercostal space in the medio-clavicular line (Electrode 1 and 2, See Fig. 1) and supra-umbilical (Electrode 4, See Fig. 1) and bilaterally infra-umbilical next to the rectus abdominis muscle (Electrode 3 and 5, See Fig. 1). Sensor signals recorded accelerations continuously in all three dimensions (x, y, z axis) each and plotted using the software MATLAB (MathWorks, Natick, MA, USA).

### Sensor specifications

The sampling rate was set to 100 Hz. Single pre-processing included a high-pass filtering with a cut-off at 0.16 Hz and a low-pass filtering set to 25 Hz. Including an A/D conversion of a 100 Hz sampling rate and a 12 bit resolution, the system achieved a resolution of 0.01 g with a sensitivity of 800 mV/g. Every three-axis-accelerometer sensor had a diameter of 5 mm and a weight of 1.5 g. The recorded signals in x, y and z direction (each axis in 7 channels) were further processed post-hoc using MATLAB software (MathWorks, Natick, MA, USA). For differentiation of thoracic and abdominal breathing components, respective sensor positions with the best signal-to-noise ratio were chosen for each case. By doing so, 6 out of 21 channels were preselected (2 times x, y and z axis) representing one thoracic and one abdominal movement vector ${\boldsymbol{x}}_{\boldsymbol{Th}}$ and${\boldsymbol{x}}_{\boldsymbol{Ab}}$ **.**

Since the direction of movement could not be analysed consistently over all individuals, only the magnitudes were considered for each sensor: $\left|{\boldsymbol{x}}_{\boldsymbol{Th}}\right|$ and $\left|{\boldsymbol{x}}_{\boldsymbol{Ab}}\right|$. Maxima and minima, which are related to respiratory activity and stand for end-inspiration and end-expiration, were identified. By calculating the gradient during inspiration phase, absolute parameters W_Th_ and W_Ab_ for breathing workload were derived – for the thoracic versus the abdominal breathing component.

### Measurement procedure

The volunteers were turned into a 30-degree elevated supine position and then connected to the clinical monitor equipment to continuous record non-invasive arterial blood pressure, breathing rates, tidal volumes, peak airway pressures and end-tidal CO_2_ concentrations. The nasal airway was occluded using a nose clip. Initially the volunteers had to breathe for 2 min without airway occlusion to record the physiological breathing patterns of the thoracic and abdominal wall. For the first workload level the participants had to breathe through an orally administered endotracheal tube of 5 mm internal diameter for 2 min. This period was followed by a second workload level of 2 min with an orally placed endotracheal tube of 4 mm internal diameter and at the third workload level subsequently through a 3 mm diameter tube. They were asked to breathe 2 min with every tube (“completed”) or until they were not able to tolerate the increasing airway resistance anymore (“abandoned”). The volunteers were asked to sustain their initial breathing frequency. We aimed to minimize the tube- changing intervals. At the end of every measurement airway occlusion pressures were recorded via the ventilator.

### Statistical analysis

Comparisons between groups for each dependent variable were made by a two-way analysis of variance (ANOVA), followed by a Tukey’s post hoc test. We formally tested normality and homogeneity of variance of the residuals by applying Shapiro-Wilk’s test and Levene’s test, respectively, with α = 0.05. If any of these two assumptions was violated, we performed a Kruskal-Wallis test followed by a Dunn’s post hoc test instead.

Data are shown as means ± SD or ± CI, when appropriate. All statistical tests are two-tailed. In all cases, a level of *p* < 0.05 was considered as statistically significant. All data were statistically analysed using a commercially available software package (GraphPad Prism 6.0, Graphpad Software Inc., San Diego, CA, USA; SPSS 21, IBM, USA).

## Results

Fifteen enrolled subjects did successfully complete the experiment (“completed” group), but 14 study participants were not able to complete the entire trial due to intolerance of increasing airway resistance caused by the decreasing tube sizes (“abandoned” group).

### Clinical measurements

**Fig. 2:**
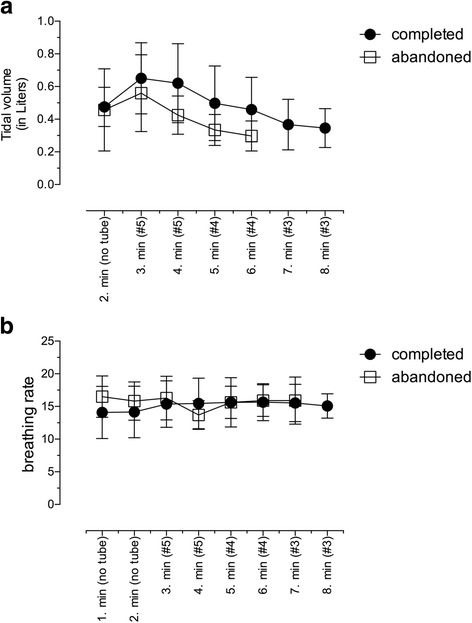
Measured time course of breathing rates (**a**) and tidal volumes (**b**) for subjects, which completed the trial (closed circles) and for subjects, which abandoned the trial (open squares). Values are displayed as means ± standard deviation

**Fig. 3:**
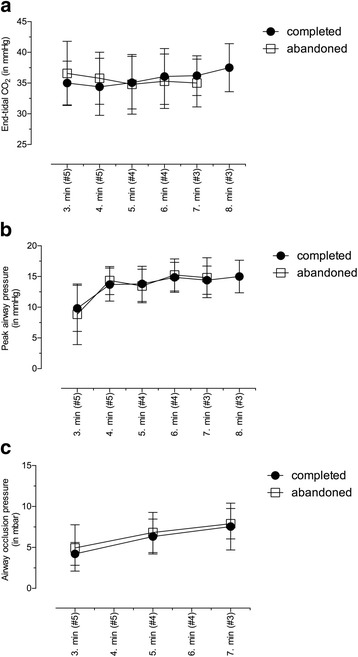
Measured time course of end-tidal CO2 (**a**), peak airway pressure (**b**) and airway occlusion pressure (**c**) for subjects, which completed the trial (closed circles) and for subjects, which abandoned the trial (open squares). Values are displayed as means ± standard deviation

Breathing frequencies of all enrolled subjects remained stable during the entire experiments and did not differ between the groups (Fig. 2a). Tidal volumes remained in physiological ranges without any group-specific difference (Fig. 2b). End-tidal CO_2_ levels were unaltered during the experimental time and were stabilized in physiological ranges (Fig. 3a).

Due to the stepwise reduction of internal tube diameters the peak airway pressure increased during the experiments, but there were no significant differences between both groups (Fig. 3b).

We did not observe any significant changes in the repeated measurements of airway occlusion pressures (Fig. 3c) and the values were comparable for both groups.

In accordance to the Hagen-Poiseuille-Law the calculated relative flow resistance of the used 5 mm tube was 1.6 N*S/m^5^, for the 4 mm tube 3.91 N*S/m^5^, for the 3 mm tube 8.87 N*S/m^5^ and for the 2 mm tube 44.87 N*S/m^5^.

### Measurements of breathing excursion

**Fig. 4:**
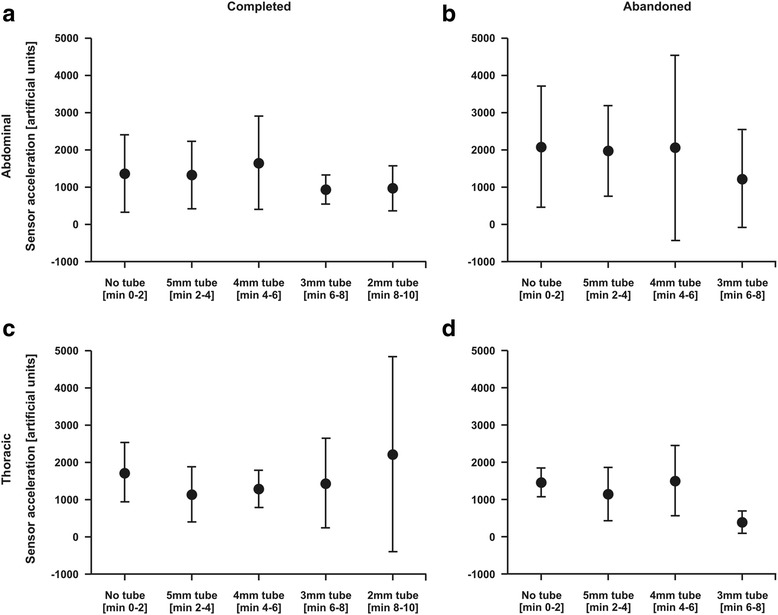
Measured acceleration difference between inspiration and expiration in the “completed” group and the “abandoned” group in the first 2 min without tube connection, from minute 2 to minute 4 orally connected to a endotracheal tube of 5 mm internal diameter, from minute 4 to minute 6 connected to a 4 mm tube, from minute 6 to minute 8 connected to a 3 mm tube and from minute 8 to minute 10 connected to a 2 mm tube. **a** displays acceleration sensor measurements in abdominal wall position of the “completed” group. **b** displays acceleration sensor measurements in abdominal wall position of the “abandoned” group. **c** displays acceleration sensor measurements in thoracic wall position of the “completed” group. **d** displays acceleration sensor measurements in thoracic wall position of the “abandoned” group. Data are displayed as means of artificial units ± CI

Gradual increased respiratory workload led to a significant decrease of acceleration in abdominal wall position in the “abandoned” group compared to the “completed” group (*p* < 0.001), while these gradual accelerating changes were not observed in thoracic wall position (*p* = 0.484) (Fig. 4). This effect was time-depend in abdominal wall position, directly correlated to the progressive gradient of respiratory workload, but missed significance (*p* = 0.054) (Fig. 4).

Thoracic acceleration sensors did not detect any time- and group-specific changes (*p* = 0.746) (Fig. 4).

## Discussion

Our study introduces the possible feasibility of acceleration sensors in abdominal wall position as a non-invasive, economical and practical approach to detect early breathing alterations prior to respiratory failure.

### A brief discussion of these findings follows

The aim of this study was to evaluate the excursion of the thoracic and abdominal wall in dependence of the airway resistance. The participating healthy volunteers did not show any significant difference in the peri-interventional registered clinical basic parameters, and therefore the collected data are considered to be comparable. As synchronic interaction of respiratory muscles, namely the diaphragm and the external and internal intercostal muscles, and the auxiliary respiratory muscles, where required, is crucial to breathing physiology.

In contrast to recent and/or established methods to assess the respiratory muscle function we investigated a non-invasive, economical and practical approach that demonstrates the valid detection of breathing excursions by accelerator sensors in presence of gradually increased airway resistance. The regressive acceleration induced by an increased respiratory workload was assessable in abdominal wall position of the sensors, but not in the thoracic position. These alterations may be indicative for the onset of reverse breathing. As inverted breathing is a well-known indicator for the onset of respiratory failure our findings in the abandoned group may be interpreted in this context, although all participants in the abandoned group ended the trail to subjective intolerance of the increased respiratory workload. Our results indicate that this effect may become perceivable before the onset of respiratory failure and can first be detected in abdominal wall position.

The activation of the abdominal muscles during expiration is known as a sign of respiratory muscle dysfunction [17], that was not expected in the healthy volunteers of this study. In our measurements, we also found no movement in the thoracic wall during increase workload. A possible explanation of this fact could be a different force of the thoracic wall compared with the abdominal wall during the attempt to compensate an increased respiratory resistance. It could be suggested that the breathing support by abdominal wall muscles has a higher impact than the thoracic muscles. As manifestation of weaning failure should be prevented, several predictive methods are used prior to its occurrence. The rapid shallow breathing index is a well-described predictor of weaning failure, but its sensitivity and specificity is controversially discussed [18–20] and vulnerable to ventilator settings and patient populations [21, 22]. Therefore our study may contribute important insight to the pathophysiological alterations prior to the onset of respiratory failure. Nevertheless, the predictive value of the measured changes by used accelerometer sensors in the context of respiratory failure needs further studies.

### Limitations

Our study has several limitations, which need to be addressed. First of all, our data were collected on healthy and alert volunteers, that breathed spontaneously, so mechanistic and central nervous alterations of respiratory function are not directly applicable to the diversity of intensive care patients in need of weaning from the ventilator. Therefore the measured changes could vary in certain populations of critical ill patients.

Further limitations like the influence of different ventilator settings, chronic pulmonary diseases, or specific surgical approaches, like cardiac or thoracic surgery, or the duration of mechanical ventilation could have an impact on the measured accelerations. Especially different surgical approaches, thoracic or abdominal surgery, may have a significant independent impact on the ratio of abdominal to thoracic breathing excursion and may differ from our findings in healthy volunteers, who did not undergo any surgery.

Moreover, there are inter-individual differences in ratio of abdominal to thoracic excursions during physiological breathing patterns, which make further investigations necessary.

Especially for patients at risk of weaning failure further studies are needed to address the value of this predictive approach.

## Conclusion

Acceleration sensors in abdominal wall position might be a feasible technique as a non-invasive, economical and practical approach to detect early breathing alterations prior to subjective intolerance of increased airway resistance.

## Abbreviations

2-way ANOVA: Two-way Analysis of Variance
RSBI: Rapid shallow breathing index

## References

[1] Murciano D, Rigaud D, Pingleton S, Armengaud MH, Melchior JC, Aubier M (1994). Diaphragmatic function in severely malnourished patients with anorexia nervosa. Effects of renutrition. Am J Respir Crit Care Med.

[2] Gea J, Casadevall C, Pascual S, Orozco-Levi M, Barreiro E (2012). Respiratory diseases and muscle dysfunction. Expert review of respiratory medicine.

[3] Fitting JW (1991). Respiratory muscle fatigue limiting physical exercise?. Eur Respir J.

[4] Bellemare F, Bigland-Ritchie B (1987). Central components of diaphragmatic fatigue assessed by phrenic nerve stimulation. Journal of applied physiology (Bethesda, Md. : 1985).

[5] Demoule A, Clavel M, Rolland-Debord C, Perbet S, Terzi N, Kouatchet A (2016). Neurally adjusted ventilatory assist as an alternative to pressure support ventilation in adults: a French multicentre randomized trial. Intensive Care Med.

[6] Tobin MJ, Perez W, Guenther SM, Semmes BJ, Mador MJ, Allen SJ (1986). The pattern of breathing during successful and unsuccessful trials of weaning from mechanical ventilation. Am Rev Respir Dis.

[7] Doorduin J, Nollet JL, Roesthuis LH, van Hees HWH, Brochard LJ, Sinderby CA, et al. Partial neuromuscular blockade during partial Ventilatory support in sedated patients with high tidal volumes. Am J Respir Crit Care Med. 2016:rccm.201605–1016OC. 10.1164/rccm.201605-1016OC. 10.1164/rccm.201605-1016OC 27748627

[8] Powers SK, Wiggs MP, Sollanek KJ, Smuder AJ (2013). Ventilator-induced diaphragm dysfunction: cause and effect. Am. J. Physiol. Regul. Integr. Comp. Physiol..

[9] Reid WD, Huang J, Bryson S, Walker DC, Belcastro AN (1994). Diaphragm injury and myofibrillar structure induced by resistive loading. J. Appl. Physiol.(Bethesda, Md. : 1985).

[10] Orozco-Levi M, Lloreta J, Minguella J, Serrano S, Broquetas JM, Gea J (2001). Injury of the human diaphragm associated with exertion and chronic obstructive pulmonary disease. Am J Respir Crit Care Med.

[11] Rácz GZ, Gayan-Ramirez G, Testelmans D, Cadot P, de Paepe K, Zádor E (2003). Early changes in rat diaphragm biology with mechanical ventilation. Am J Respir Crit Care Med.

[12] Pettersen V (2005). Muscular patterns and activation levels of auxiliary breathing muscles and thorax movement in classical singing. Folia Phoniatr. Logop.: official organ Int. Assoc. Logoped. Phoniatr. (IALP).

[13] McCool FD, Loring SH, Mead J (1985). Rib cage distortion during voluntary and involuntary breathing acts. J. Appl. Phys. (Bethesda, Md. : 1985).

[14] Blankman P, Gommers D (2012). Lung monitoring at the bedside in mechanically ventilated patients. Curr Opin Crit Care.

[15] Tobin MJ (2006). Remembrance of weaning past: the seminal papers. Intensive Care Med.

[16] Talmor D, Sarge T, Malhotra A, O'Donnell CR, Ritz R, Lisbon A (2008). Mechanical ventilation guided by esophageal pressure in acute lung injury. N Engl J Med.

[17] Parthasarathy S, Jubran A, Laghi F, Tobin MJ (2007). Sternomastoid, rib cage, and expiratory muscle activity during weaning failure. J. Appl. Phys. (Bethesda, Md : 1985).

[18] Verceles AC, Diaz-Abad M, Geiger-Brown J, Scharf SM (2012). Testing the prognostic value of the rapid shallow breathing index in predicting successful weaning in patients requiring prolonged mechanical ventilation. Heart & lung : J. Crit. Care.

[19] Danaga AR, Gut AL, Antunes LC d O, Ferreira ALDA, Yamaguti FA, Christovan JC (2009). Evaluation of the diagnostic performance and cut-off value for the rapid shallow breathing index in predicting extubation failure. J. Bras. Pneumol.: publicacao oficial da Sociedade Brasileira de Pneumologia e Tisilogia.

[20] Karthika M, Enezi A, F A, Pillai LV, Arabi YM (2016). Rapid shallow breathing index. Annals of thoracic medicine.

[21] Smailes ST, McVicar AJ, Martin R (2013). Cough strength, secretions and extubation outcome in burn patients who have passed a spontaneous breathing trial. Burns : journal of the International Society for Burn Injuries.

[22] Boutou AK, Abatzidou F, Tryfon S, Nakou C, Pitsiou G, Argyropoulou P, Stanopoulos I (2011). Diagnostic accuracy of the rapid shallow breathing index to predict a successful spontaneous breathing trial outcome in mechanically ventilated patients with chronic obstructive pulmonary disease. Heart & lung : J. Crit. Care.

